# Enzymic Activity, Metabolites, and Hematological Responses in High-Risk Newly Received Calves for “Clinical Health” Reference Intervals

**DOI:** 10.3390/ani14162342

**Published:** 2024-08-14

**Authors:** Octavio Carrillo-Muro, Daniel Rodríguez-Cordero, Pedro Hernández-Briano, Paola Isaira Correa-Aguado, Carlos Aurelio Medina-Flores, Luis Arturo Huerta-López, Francisco Javier Rodríguez-Valdez, Alejandro Rivera-Villegas, Alejandro Plascencia

**Affiliations:** 1 Unidad Académica de Medicina Veterinaria y Zootecnia, Universidad Autónoma de Zacatecas, General Enrique Estrada 98500, Mexico; octavio_cm@uaz.edu.mx (O.C.-M.); paocorrea@uaz.edu.mx (P.I.C.-A.); carlosmedina@uaz.edu.mx (C.A.M.-F.); huerta_lopez@uaz.edu.mx (L.A.H.-L.); franciscorv@uaz.edu.mx (F.J.R.-V.); alejandro.rivera@uaz.edu.mx (A.R.-V.); 2 Facultad de Medicina Veterinaria y Zootecnia, Universidad Autónoma de Sinaloa, Culiacán 80260, Mexico; alejandro.plascencia@uabc.edu.mx

**Keywords:** beef calf, blood cells, calcium, electrolytes, glucose, platelets, enzymic, lipids, protein

## Abstract

**Simple Summary:**

Enzymic activity, metabolites, and hematological responses for reference intervals (RIs) establish ranges of physiological normality, which are useful to diagnose diseases and physiological alterations. The initial days after calves arrive at the feedlot are the most critical because several stressors affect immunological and metabolic responses of high-risk newly received calves. Surprisingly, no reports determine RIs for different enzymic activity, metabolites, and hematological responses in high-risk newly received calves during this stage. This study is the first to establish RIs for different enzymic activity, metabolites, and hematological responses in high-risk newly received calves in this period. This information will be useful for veterinary clinical practice and research related to the health and welfare of high-risk newly received calves during their initial period at a feedlot.

**Abstract:**

Enzymic activity, metabolites, and hematological responses for reference intervals (RIs) establish ranges of physiological normality, which are useful for diagnosing diseases and physiological alterations. Within the same species, RIs vary according to age, gender, productive and physiological states, and environmental factors including health management and nutrition. RIs have been extensively studied in dairy calves during a critical stage of life (from birth up to first 90 days of age). A critical stage for feedlot calves is their arrival at the feedlot, but no reports determine RIs for different enzymic activity, metabolites, and hematological responses during their initial period at the feedlot. Consequently, a total of 461 high-risk crossbreed beef calves, received on three different dates, were examined upon arrival at the feedlot. Of these, 320 calves (148.3 ± 1.3 kg body weight) whose “clinical health” was evaluated were included in the study. Blood samples were taken upon arrival and on days 14, 28, 42, and 56 to determine the following parameters: enzymic activity, metabolites, electrolytes, white blood cells, platelets, and red blood cells. Enzymic activity, metabolites, and complete blood count were determined by automated analyzers. The freeware Reference Value Advisor Software was used to calculate the non-parametric values of RIs. This study is the first to establish RIs for different enzymic activity, metabolites, and hematological responses in high-risk newly received calves during their initial period at the feedlot. This information will be useful for veterinary clinical practice and research related to the health and welfare of high-risk newly received calves during their initial period at the feedlot.

## 1. Introduction

The health and management of newly received cattle continue to pose significant animal welfare and economic challenges for the beef industry. These calves are subjected to several stressors such as recent weaning [1,2], castration [3], transportation [4,5], commingling of cattle from various sources [6,7], and adaptation to new environmental surroundings and novel feed ingredients [8]. All these factors predispose calves to increased susceptibility to digestive disturbances and respiratory diseases, raising morbidity and mortality rates in the feedlot at this stage [9,10]. Therefore, these calves are classified as “high-risk calves”. Since the immune system is compromised in high-risk calves, they are more vulnerable to infectious agents [11]. As a result, morbidity rates can increase by 16.2% and mortality rates rise during the reception period (first 42 or 56 days) [8,12]. This is significant since 28% of cattle placed in North American feedlots are high-risk cattle [13]. 

Correctly evaluating the health status of newly received calves is essential for applying accurate medical treatments or strategies to minimize the risk of aggravated clinical symptoms. Enzymic activity, metabolites, and hematological responses for reference intervals (RIs) establish ranges of physiological normality useful for diagnosing diseases and physiological alterations; therefore, RIs are invaluable for determining physiological, nutritional, metabolic, and clinical status [14,15]. 

Despite the fact that some studies present data on enzymic activity, metabolites, and hematological responses in high-risk newly received calves concerning their “clinical health”, these are not useful as RIs. Most research on high-risk newly received calves has been conducted for other purposes (inferential statistics) using a reduced number of observations, and the data presented are only mean values with their standard errors [16,17,18,19,20,21]. To determine whether certain enzymic activity, metabolites, and hematological responses are within normal ranges, a comparison with the RIs of a similar population of clinically healthy individuals is required [22,23]. Therefore, RIs must be specifically established for particular conditions; in this case, for high-risk newly received calves’ “clinical health”.

Several studies have determined enzymic activity, metabolites, and hematological responses in beef cattle at various production stages and within different production systems. These studies have been developed for pre-weaning calves [5,24,25], for adult cattle in a feedlot [26,27], and for cattle under extensive production systems with calves from the same origin [20]. However, surprisingly, there are no reports determining RIs for different enzymic activity, metabolites, and hematological responses of high-risk newly received calves’ “clinical health”.

Due to the special conditions of calves recently arriving at the feedlot, using published RIs values as references can lead to imprecise diagnoses. Therefore, it is necessary to establish robust and specific RIs for this type of cattle to adequately discriminate between healthy and sick calves during their initial period at the feedlot.

For this reason, the aims of this study were: (1) determining RIs in high-risk newly received calves whose “clinical health” was evaluated related to enzymic activity, metabolites, and hematological responses; and (2) to compare these RIs with published values of enzymic activity, metabolites, and hematological responses in high-risk newly received calves during their initial period at the feedlot. 

## 2. Materials and Methods

### 2.1. Location Where the Study Was Performed

This study was conducted at a private stocker facility, Torunos Livestock Preconditioning Center, within the experimental area in Fresnillo, Zacatecas, Mexico (23°08′56.22″ N and 102°43′48.57″ W), owned by Grupo Exportador Pa Lante S.P.R. de R.L. The area, at an elevation of approximately 2081 m above sea level, experiences a semi-dry temperate climate. Sample collections were performed on the farm and processed in the Laboratorio de Análisis Clínicos Veterinarios of the Unidad Académica de Medicina Veterinaria y Zootecnia at the Universidad Autónoma de Zacatecas (UAMVZ-UAZ).

Sampling was carried out on three different dates, with the following average ambient air temperatures: (1) June–July 2023: 22.4 °C (minimum of 12.2 °C and a maximum of 28.3 °C); (2) November–December 2023: 14.3 °C (minimum of 6.1 °C and a maximum of 19.8 °C); and (3) April–May 2024: 20.1 °C (minimum of 9.6 °C and a maximum of 33.1 °C). Total precipitation was 365.0 mm, and the average relative humidity was 40%. 

### 2.2. Ethics Statement

This research was approved by the UAMVZ-UAZ—Institutional Bioethics and Animal Welfare Committee (Protocol #2023/05/16). The research was conducted in compliance with the Official Mexicans Standards: (1) NOM-051-ZOO-1995, Humanitarian care in the mobilization of animals; (2) NOM-062-ZOO-1999, Humane care and welfare of experimental animals; and (3) NOM-024-ZOO-1995, Animal health stipulations and characteristics during animal transportation. The owner of Grupo Exportador Pa Lante S.P.R. de R.L. gave written, informed consent for the animals to take part in the research.

### 2.3. Inclusion Criteria

The calves came from a system of production of dual-purpose, cow-calf, under conditions of extensive grazing, where they were weaned with a weight range of 100 to 200 kg and were taken for sale to the livestock collection center in Milpillas de la Sierra, Valparaiso, Zacatecas. They were kept in the place for no more than 14 days until the day of their transfer. Cattle with the following characteristics were considered high-risk newly received calves: (1) unknown health and management background; (2) weight at arrival between 150 and 200 kg; (3) age between 5 and 7 months; (4) weaned for a maximum of 14 d; (5) exposed to handling and transportation; (6) commingled with calves from different sources, and (7) unvaccinated upon arrival to feedlot. Additionally, calves included in the first sampling had to meet the following criteria within 24 h of arrival: be cross beef cattle (without dairy breed crosses), be evaluated as clinically healthy by an experienced veterinarian (considering alertness, vigor, absence of dehydration, diarrhea, cough, and nasal or ocular discharge), present physiological parameters within normal ranges (cardiac auscultation >86 and <125 beats per minute; normal lung auscultation >20 and <44 breaths per minute; normal rectal temperature >38 and <39.5 °C; normal mucosal color), and not have received antibiotic or anti-inflammatory therapy at the livestock collection center.

### 2.4. Cattle Processing 

The animal health protocols and arrival procedures were established by the cooperating producer and were not altered for this study. High-risk bull calves (*n* = 461) were sourced regionally, grouped at an order buying facility in Milpillas de la Sierra, and transported approximately 4 h (120 km) to the Torunos Livestock Preconditioning Center, shipped in two Kenworth cattle hauling model 2014 vehicles, with a space of 2 m^2^/calf. Calves arrived on three dates: 1 June 2023 (*n* = 137), 6 November 2023 (*n* = 166), and 11 April 2024 (*n* = 158). Calves received on each date were randomly divided into two pens (50 or 60 calves/pen), ensuring that each blood sampling period (0, 14, 28, 42, and 56 d) was replicated three times (once each date). 

Upon arrival, calves were provided with free-choice oat hay, water, and mineral supplements for 12 h. The following morning (0600 h), calves were processed as follows: (1) metaphylactic treatment with oxytetracycline (5 mg/kg BW; Emicina^®^ líquida, Zoetis, Ciudad de Mexico, Mexico); (2) injection with 5 mL vaccination for clostridial organisms (Biovac 11 Vías^®^, Biozoo, Jalisco, Mexico); (3) treatment for control of internal (4% ivermectin, Master LP^®^ injectable, Ourofino Salud Animal, São Paulo, Brazil) and external parasites (pour on cypermethrin, Cypermil Pour On^®^, Ourofino Salud Animal, São Paulo, Brazil); (4) application of a sequentially numbered identification tag in the left ear, recording the presence of any existing ear tags; and (5) recording individual initial body weight (IBW, electronic scale; Metrology^®^ PBG-3000, cap. 2000 kg, Básculas y Accesorios de Peso SA de CV, Nuevo Leon, Mexico; readability 0.454 kg). Of the 461 calves received, a total of 320 calves met all inclusion criteria. The distribution of included calves per date was: 1 June 2023: *n* = 100; 6 November 2023: *n* = 120; and 11 April 2024: *n* = 100). On average, the calves weighed 148.3 ± 1.3 kg BW, were 5.5 months old, and had a rectal temperature of 38.7 °C.

### 2.5. Feeding and Health Management

The selected calves from each arrival date were placed in two soil-surface outdoor pens (50–60 calves/pen) where they remained for 56 days. Calves were fed a receiving diet formulated to meet the nutrient requirements specified by NASEM [28]. The estimated nutrient composition of the receiving diets was: dry matter, 86.2%; crude protein (CP), 14.6%; ether extract, 4.6%; neutral detergent fiber, 29.9%; net energy for maintenance, 1.69 Mcal/kg; and net energy for gain, 1.08 Mcal/kg. Calves were fed a 50% concentrate diet from day 0 to day 14. The concentrate proportion was increased on day 15 and day 28 (to 60 and 70% concentrate diets, respectively). The 70% concentrate diet was fed for the remainder of the trial (day 29 to day 56). Feed was offered at 90% of the amount delivered the previous day on each transition day. Calves were provided fresh feed three daily at 0800, 1200, and 1600 h. The amount of feed given at 0800 and 1200 h remained constant, while the feed offered at 1600 h was adjusted daily to ensure a residual feed of approximately ~100 g/calf. Calves were observed daily by an experienced veterinarian for any signs of bovine respiratory distress, including labored breathing, nasal or ocular discharge, depression, anorexia, lethargy, or abnormal appetites. According to the pen’s disease monitoring (mentioned in Section 2.4) and medical treatments protocols of the feedlot, no sick animals were detected during the study period.

### 2.6. Assessment of Enzymic Activity, Metabolites, and Hematological Responses

**Figure 1:**
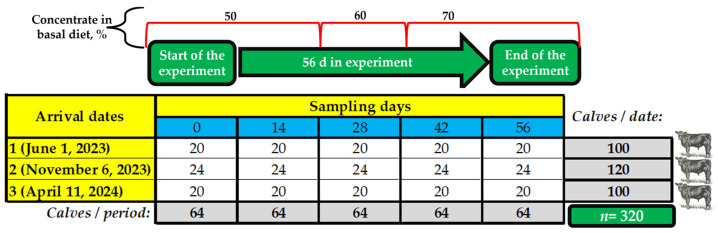
Arrival dates (3) and sampling schedule performed at day 0, 14, 28, 42, and 56 of arrival to feedlot (5 samplings).

Blood samples (10 mL) were taken on days 0, 14, 28, 42 and 56 from the arrival. A total of 20% of calves from each arrival date was randomly selected for each sample day, resulting in a total of 320 blood samples at the end of sampling (Figure 1). For blood sample collection, calves were restrained in a standing position with the help of squeeze chutes (Priefert^®^, Model S0191, Mount Pleasant, TX, USA). Blood was collected by the same experienced veterinarian each time before morning feeding (approximately 0700 h) by jugular venipuncture using 18-gauge × 3.81 cm, after preparing the venipuncture site with a gauze swab soaked in 70% alcohol.

Whole blood samples were collected using a 5.0 mL BD Vacutainer, while blood samples for serum were collected in a 5.0 mL BD Vacutainer SST and centrifuged (2500× *g* at 4 °C for 15 min) within 15 min of collection. After collection, whole blood and serum samples were stored in coolers (4 °C) and transported directly to the university laboratory, approximately 40 km away. Upon arrival, samples were immediately analyzed (within 2 h of collection).

Whole blood samples were analyzed for a complete blood count using an automatic cell counting machine (Exigo veterinary haematology analyser^®^, Boule Medical AB, Spånga, Sweden). The following parameters were determined: total white blood cells (WBC), lymphocytes (LYM), lymphocytes % (LYM%), monocytes (MON), monocytes % (MON%), granulocytes (GRA), granulocytes % (GRA%), platelets (PLT), mean platelet volume (MPV), red blood cells (RBC), RBC distribution width test % (RDW%), hemoglobin (HGB), hematocrit (HCT), mean corpuscular volume (MCV), mean corpuscular HGB (MCH), and mean corpuscular HGB concentration (MCHC).

Enzymic activity and metabolites were quantified using an automated analyzer (FUJI DRI-CHEM NX500^®^; Fujifilm, Tokyo, Japan). The following parameters were determined: activity of alkaline phosphatase (ALP), gamma glutamyltransferase (GGT), aspartate aminotransferase (AST), and alanine aminotransferase (ALT); levels of total protein (TP), albumin (ALB), globulin (GLO = TP − ALB, [29]), blood urea nitrogen (BUN), creatinine (CRE), total bilirubin (TBIL), total cholesterol (TCHO), triglycerides (TG), calcium (Ca), glucose (GLU), sodium (Na^+^), potassium (K^+^), and chlorine (Cl^−^). 

### 2.7. Calculation of Reference Intervals

The number of high-risk bull calves enrolled in this study was based on guidelines from the American Society for Veterinary Clinical Pathology [30]. RIs were calculated using Reference Value Advisor Software (v.2.1) freeware (RefValAdv), an add-in program in Excel (Office 365, Microsoft, Redmond, WA, USA) [31]. The RefValAdv output displays descriptive statistics, histograms, and Q/Q plots for each variable and calculates 95% RIs with 90% confidence intervals (CI), based on normality and symmetry of data distribution, outliers, and sample size. For population sizes over 120, non-parametric RIs were calculated directly. Normality and distribution tests were not required for these variables [32], though histograms for all variables were visually inspected for evidence of any unusual or unexpected distributions or data points. The criteria for outlier removal were as follows: (1) For interquartile range (IQR) = Q3(third quartiles) − Q1(first quartiles), values that exceed interquartile fence set at Q1 – 3 × IQR and Q3 + 3 × IQR are considered outliers; (2) If Reference Value Advisor computes results with a good distribution and/or symmetry without removing certain outliers, then the outliers are retained; and (3) If no normal distribution and/or symmetry are observed even after outliers are removed and data are power transformed, the population is calculated with Cook’s distance and Cook’s outliers are detected and deleted. After the removal of outliers, populations were recalculated for RIs. Finally, comparisons between our means and those of other publications of high-risk newly received calves “clinical health” were performed with the *t*-test, using PROC TTEST in SAS^®^ software 9.3, Cary, NC, USA.

## 3. Results and Discussion

The averages obtained in this study, when compared with those reported in other publications on enzymic activity, metabolites, and hematological responses, were statistically different for most variables (*p* < 0.05). The differences that were observed compared to other authors could be explained by the following factors: (1) Different sample sizes (*n*): 10 [21], 12 [18], 24 [16], 31 [20], or 48–72 [19], with experimental units of the treatment considered as the control, and in some cases, *n* was not reported [17]; (2) Different IBW: 147 ± 1.67 kg [21], 220 ± 16.2 kg [16], 252 ± 5.5 kg [17], 258 ± 22.5 kg [19], and in some cases this variable was not reported [18]; (3) Different sex: bulls [16,21], steers [19], or mixed [17]; (4) Production systems: feedlot [16,17,19,21] or extensively cultivated pastures [20]; and (5) Different geographical locations: Zacatecas State, México [21], Texas State, USA [16,17,19], Mississippi State, USA [18], or Tocantins State, Brazil [20]. However, despite all these factors and the observed differences between the analyzed averages, most of them are within the RIs generated in this research. Therefore, these RIs for enzymic activity, metabolites, and hematological responses are relevant and useful as a diagnostic tool for determining the physiological, nutritional, metabolic, and clinical status of high-risk newly received calves “clinical health”, regardless of different IBWs, sexes, production systems, and geographical locations.

### 3.1. Enzymic Activity

Table 1 shows the descriptive statistics and RIs generated for the different enzymic activity and metabolites; and in Table 2, based on the means generated in Table 1, a comparison is made with means from different publications that also studied high-risk newly received calves’ “clinical health”.

The quantification of enzymic activity in high-risk newly received calves aims to determine if there is any pathological liver or kidney damage or any changes in the metabolism of these organs [33,34]. The means from this study and those reported by different authors [16,17,21], are within the RIs generated for ALP (14.4–469.6), GGT (10.0–47.90), AST (38.9–124.2), and ALT (16.6–44.4). ALP activity values were 209.70, lower than those observed by Rodríguez-Cordero et al. [21] (379.40, *p* < 0.001), but higher than those reported by Smock et al. [19] (111.36, *p* < 0.001); elevated activity values are related to bone growth and osteoblast proliferation in young cattle [35]. Regarding GGT activity, values were 17.00, similar to those observed by Rodríguez-Cordero et al. [21] (16.60, *p* > 0.05); increases are attributed to enhanced liver activity [36]. Serum AST activity values were 68.80, comparable to Smock et al. [19] (69.38, *p* > 0.05), but lower than those reported by Rodríguez-Cordero et al. [21] (74.70, *p* < 0.001). AST is an enzyme associated with highly metabolic tissues, primarily cardiac muscle, liver cells, and skeletal muscle cells. AST increases when an infection or injury causes the lysis of cells in these tissues, releasing AST into the blood [16,37]. Therefore, AST is a nonspecific indicator of tissue damage and can be elevated in cases of muscle injury or necrosis, particularly in recumbent animals [33,38]. Thus, dietary deficiencies in muscle-related nutrients such as vitamin E or selenium can also cause muscular dystrophy, and the diagnosis is generally based on elevated AST levels [39]. Finally, ALT activity is commonly used to assess hepatocellular injury in small animals, but it is low in ruminants and, therefore, not useful for evaluating liver function [29]; the ALT activity value was 26.50, higher than reported by Crawford et al. [17] (*p* < 0.001, 16.92) and Smock et al. [19] (*p* < 0.001, 19.55).

### 3.2. Metabolites

In calves, enzymic activity and metabolite concentrations can indicate real-time nutritional status [40] and the efficiency with which they utilize dietary nutrients [41].

#### 3.2.1. Total Protein, Blood Urea Nitrogen, and Creatinine

Proteins perform very specific functions in the body; however, there is no single metabolite that directly indicates the protein status. Therefore, it is necessary to determine a series of metabolites to understand the protein status of the animal, such as TP (ALB and GLO), BUN, and CRE. The means from this study and those reported by different authors [16,17,21], fall within the RIs generated for TP (4.4–7.7), ALB/GLO ratio (0.68–1.32), BUN (6.91–16.1), and CRE (0.52–1.35). However, the means reported by Smock et al. [19] are within (2.45) of the RIs generated for ALB (1.90–3.70), but those observed by Rodriguez-Cordero et al. [21] were slightly higher (3.90). Similarly, Rodriguez-Cordero et al. [21] report values within (3.10) of the RIs generated for GLO (2.20–4.11), but those observed by Smock et al. [19] were higher (4.50–4.11). 

Serum TP, which mainly includes ALB and GLO (the primary solid components of serum), is quantified to determine the nutritional status of high-risk newly received calves [42]. TP is minimal at the start of the reception process, followed by an increase during the rest of this stage; this could indicate a low concentration of immunoglobulins (GLO) upon arrival at the feedlot, followed by development from vaccination on day 28 or from a natural infection [16]. The TP value was 6.22, higher than reported by Rodríguez-Cordero et al. [21] (5.90, *p* < 0.001), but lower than Smock et al. [19] (6.96, *p* < 0.001); normal values indicate adequate availability and digestibility of microbial protein [27,38,43]. Regarding ALB, the value was 2.97, above those reported by Smock et al. [19] (2.45, *p* < 0.001), but lower than those observed by Rodríguez-Cordero et al. [21] (3.90, *p* < 0.001); high concentrations within RIs are associated with growing animals, suggesting proper nutrition [43]. GLO concentrations were 3.18, higher than those reported by Rodríguez-Cordero et al. [21] (3.10, *p* < 0.05), but were lower than those observed by Smock et al. [19] (4.50, *p* < 0.001). Elevated concentrations can result from high CP intake, and normal concentrations are related to a lower likelihood of significant chronic bacterial infection [34]. The presence of hyperproteinemia results from increased concentrations of ALB, GLO, or both. The only cause of hyperalbuminemia is dehydration; when dehydration occurs, ALB and GLO levels increase. However, if there is hyperproteinemia without concurrent dehydration, it is generally the result of hyperglobulinemia. Common causes of hyperglobulinemia include chronic antigenic stimulation (i.e., chronic inflammatory disease) and liver disease. Chronic antigenic stimulation is usually observed in various conditions, such as traumatic reticuloperitonitis, liver abscesses, or chronic pneumonia [29]. Finally, the RIs calculated for the ALB/GLO ratio (0.68–1.32) are slightly broader than those indicated by Kaneko et al. [27] (0.84–0.94); however, they are fairly constant in healthy cattle.

The determination of serum or blood urea nitrogen (SUN or BUN) aims to evaluate CP intake and nutrient balance [44]; it is used to estimate the efficiency in N utilization and its excretion [45]. BUN value were 11.08, similar to that observed by Rodríguez-Cordero et al. [21] (11.30, *p* > 0.05), but higher than those reported by Smock et al. [19] (*p* < 0.001, 8.22) and Crawford et al. [17] (*p* < 0.001, 8.67). Most calves in reception show lower N retention, probably due to increased muscular catabolism to obtain proteins and improve the immune response [46]; all of which is due to limited feed and water intake, as well as the stress caused by marketing and transportation [47,48,49]. Additionally, there is evidence that in high-risk newly received calves, protein catabolism is higher (elevated BUN) in the initial period of arriving at the feedlot, which could indicate that calves are releasing protein reserves to compensate for poor CP intake or inadequate CP content in the diet; as these types of animals depend more on CP from the diet than on muscular catabolism [50,51].

CRE concentrations were 0.81, higher than that observed by Rodríguez-Cordero et al. [21] (0.75, *p* < 0.001), but lower than that reported by Smock et al. [19] (*p* < 0.001, 1.19) and Crawford et al. [17] (*p* < 0.001, 1.20). The CRE value within RIs indicates that the calves have an appropriate renal glomerular filtration rate; high serum levels are indicative of nephritis [29]. On the other hand, reduced CRE concentrations indicate prolonged active catabolism of tissue proteins [52], as seen in emaciated cattle or those with little muscle mass; or being above the RIs in heavily muscled animals [29,35,36] or in animals that walk long distances in search of a pasture [38]. 

#### 3.2.2. Total Bilirubin, Total Cholesterol, and Triglycerides

TBIL, a breakdown product of HGB, is transported to the liver bound to ALB where it is conjugated and excreted into bile. In healthy cattle, TBIL concentration is low compared to other species, and its increase tends to be relatively small and inconsistent, even in severe liver disease [27]. TBIL is an important indicator of liver function, as it increases during severe lipidosis [53,54], and decreases when the liver is healthy. The concentrations of TBIL in this study and the consulted publications [16,21] are within our RIs (0.20–1.30); the value was 0.34, higher than that observed by Rodríguez-Cordero et al. [21] (0.23, *p* < 0.001) and Smock et al. [19] (0.29, *p* < 0.05).

Serum lipids mainly consist of TCHO and TG, reflecting the liver’s energy metabolism, particularly the export of lipids in the form of very low-density lipoproteins [55]. The concentrations of TCHO in this study and consulted publications [21], are within our RIs (50.0–127.7); however, they can increase in response to stressful stimuli [56] or the ingestion of energy-rich, lipid-rich feeds [57], and their decrease indicates an energy deficit. For TG values, it was 17.00, lower than that observed by Rodriguez-Cordero et al. [21] (35.10, *p* < 0.001).

#### 3.2.3. Calcium

Serum calcium is utilized in many metabolic enzymatic pathways and contributes fundamentally to muscular contraction, cardiac function, neuronal transmission, and blood coagulation. When blood Ca levels decrease, parathyroid hormone is released, stimulating the release of Ca from bone and teeth reservoirs [16,37]. Therefore, Ca is a fundamental component contributing to the development of the skeletal system in young animals; however, in adult animals, the demand for this purpose decreases, reflected in lower blood Ca levels [42]. Calcium concentrations, in this study and consulted publications [16,21], fall within our RIs (7.12–12.50); the value was 10.28, being similar to Smock et al. [19] (10.27, *p* > 0.05), but higher than that reported by Rodríguez-Cordero et al. [21] (10.10, *p* < 0.05). On the other hand, hypercalcemia is fairly rare in ruminants, usually occurring as a result of administration of Ca solutions or gels [29]. 

#### 3.2.4. Glucose

GLU and insulin are potent indicators of satiety [58,59], making their determination very useful for assessing the energy status of the animal [60] and the utilization of dietary nutrients [61]. However, their diagnostic value is moderate, as their concentration in beef cattle is influenced by adequate dry matter intake (DMI) [60] or by nutrient availability; low values are observed with low DMI [62]. It is also known that glucocorticoids widely influence GLU homeostasis in mammals [16], indicating that hyperglycemia can also occur with stress (including excitement, fear, and intense pain) [29]. In growing calves, the need for GLU is determined by the growth rate, which is influenced by the intake of metabolizable energy, whereas adult animals only require maintenance energy [63]. In calves upon arrival, the GLU is lower at the beginning of the stage and increases as the days pass; this is due to the lack of access to feed during transportation, and therefore, decreased rumination activity [16]. The GLU concentrations in this study and the consulted publications [16,21], are within our RIs (26.1–126); the value was 89.00, close to that observed by Crawford et al. [17] (90.17, *p* > 0.05), but lower than that reported by Smock et al. [19] (94.74, *p* < 0.001) and Rodríguez-Cordero et al. [21] (106.40, *p* < 0.001).

#### 3.2.5. Electrolytes

The concentration of electrolytes in the serum of high-risk newly received calves, in the present study and as reported by other authors [16,17,21], is within our RIs for Na^+^ (98.2–143), K^+^ (3.11–8.59), and Cl^−^ (71.1–109.0). Na^+^ plays a fundamental role in regulating osmotic pressure in the body, which affects the movement of water, nutrients, and waste across membranes. Another function of Na^+^ is its involvement in regulating the acid-base balance within the body [62]. Commonly, low Na^+^ values are due to diets not fully meeting nutritional requirements. The most common causes of hyponatremia are the absence or inadequate level of Na^+^ in the diet [26] or due to diarrhea [33]; the Na^+^ value was 126.30, higher than that observed by Rodríguez-Cordero et al. [21] (121.20, *p* < 0.001), but lower than that reported by Smock et al. [19] (135.82, *p* < 0.001) and Crawford et al. [17] (140.75, *p* < 0.001). Generally, alterations in Cl^−^ concentration are related to similar proportional changes in Na^+^ concentration as a result of changes in relative water balance. For example, situations such as diarrhea, blood loss, or sweating typically show a proportional decrease in Cl^−^ compared to Na^+^ [33]; the Cl^−^ value was 90.80, above that observed by Rodríguez-Cordero et al. [21] (85.70, *p* < 0.001), but lower than that reported by Smock et al. [19] (94.81, *p* < 0.001) and Crawford et al. [17] (96.88, *p* < 0.001). Lastly, K^+^ deficiency is commonly associated with cattle stress as a result of dehydration and K^+^ loss in tissues [64], but it is also frequently associated with modifications in feed intake and K^+^ absorption, as occurs in cases of diarrhea [33]. However, elevated K^+^ could reflect increased stress and shrinkage in certain individuals, potentially those rapidly weaned and transported, compared to calves weaned and exposed to a feeding regimen prior to transport [48]. The K^+^ value was 4.93, higher than that observed by Rodríguez-Cordero et al. [21] (4.40, *p* < 0.001), but lower than that reported by Smock et al. [19] (5.20, *p* < 0.001) and Crawford et al. [17] (5.70, *p* < 0.001).

### 3.3. Hematological Responses

Table 3 shows the descriptive statistics and the RIs generated for the different cells of the hemogram; and in Table 4, a comparison is made from the means generated in Table 3 with means from different publications that also studied high-risk newly received calves.

#### 3.3.1. White Blood Cells

A low concentration of WBC (leukopenia) often occurs in metabolic disorders, liver diseases, and infectious diseases [65], while an increase (leukocytosis) has been reported, generally in poisoning, bacterial infections, anaphylactic shock, stress [66], and traumatic reticuloperitonitis [66,67]. However, stressful events for high-risk beef calves upon reception, such as marketing, transportation, and arrival at the feedlot [49], induce calves to produce glucocorticoids, which promotes an increase in WBC counts [68] in the initial period of arriving [19]. On the other hand, the amount of WBC, in this study and as reported by other authors [18,19,21], is within our RIs (4.6–15.20); the concentration was 9.65, higher than that observed by Scott et al. [18] (7.32, *p* < 0.001) and Rodríguez-Cordero et al. [21] (8.90, *p* < 0.001), but lower than that reported by Smock et al. [19] (13.03, *p* < 0.001). However, a higher total WBC count may also indicate that an animal has a greater capacity to generate an innate or adaptive immune response to a foreign antigen (improved immune status), which is a key goal of vaccination [69,70]. 

The quantity of LYM, both in this study and in the consulted publications [19,21], falls within our RIs (2.6–9.0), as does the LYM% (33.6–74.61). However, it is described that moderate levels of dehydration in high-risk beef calves can increase LYM values [36,65], but it can also be due to the calves’ response to infection or inflammation, as in the case of chronic viral diseases, purulent peritonitis, cholera, mastitis, and chronic inflammation of the liver or kidneys; conversely, a low concentration may indicate viral and bacterial infections, acute stress, chronic renal failure, and after corticosteroid use [65]. LYM was 5.87, higher than that observed by Rodríguez-Cordero et al. [21] (5.10, *p* < 0.001), but lower than that reported by Smock et al. [19] (6.79, *p* < 0.001); LYM% was 59. 20, higher than that observed by Scott et al. [18] (51.63, *p* < 0.001), Smock et al. [19] (53.17, *p* < 0.001), and Rodríguez-Cordero et al. [21] (54.00, *p* < 0.001).

The amount of MON, both in this study and as reported by Rodriguez-Cordero et al. [21] and Scott et al. [18], is within our RIs (0.30–1.40), as is the % MON (5.54–12.00); however, the values reported by Smock et al. [19] were higher for these two variables (1.7 and 13.43, respectively). However, it has been reported that during situations of acute stress and in the recovery phase of acute and chronic infections, there is an increase in the number of MON, which may be caused by hemorrhage, ulcerations, exudative inflammation, necrosis, and corticosteroid therapy [71]. Likewise, a decrease in its concentration may be indicative of endotoxemia, viral diseases, and inflammation; however, it has not been demonstrated to have great clinical relevance [15,72]. MON was 0.83, higher than Rodriguez-Cordero et al. [21] (0.70, *p* < 0.001), but lower than Smock et al. [19] (1.70, *p* < 0.001); MON% was 8.28, similar to Rodriguez-Cordero et al. [21] (7.90, *p* > 0.05) and Scott et al. [18] (8.36, *p* < 0.001), but lower than Smock et al. [19] (13.43, *p* < 0.001).

The quantity of GRA, both in this study and in the consulted publications [21], falls within our RIs (1.1–6.74), as does the GRA% (17.51–85.57). GRA was 3.33, similar to that observed by Rodriguez-Cordero et al. [21] (3.10, *p* > 0.05); GRA% was 35.30, lower than that reported by Rodriguez-Cordero et al. [21] (38.00, *p* < 0.01).

#### 3.3.2. Platelets

The amount of PLT, both in this study and as reported by Motta et al. [20] and Rodriguez-Cordero et al. [21], is within our RIs (91.2–444.90), as is the MPV (6.14–9.08); however, the value reported by Smock et al. [19] for PLT were higher (513.70). 

However, thrombocytosis, or elevated PLT count, can be associated with acute or chronic infections and inflammation [19]. In clinical situations where there is severe bleeding or a higher risk of bleeding, it is considered appropriate to perform a PLT count. This includes the presence of clinical signs such as petechiae, ecchymosis, hematuria, epistaxis, melena, hematemesis, and hemarthrosis [73]. PLT was 239.80, similar to that observed by Rodríguez-Cordero et al. [21] (243.00, *p* > 0.05), but lower than that reported by Smock et al. [19] (513.70, *p* < 0.001). Regarding MPV, it was 7.22, similar to that observed by Rodríguez-Cordero et al. [21] (7.20, *p* > 0.05).

#### 3.3.3. Red Blood Cells

The quantity of RBC, both in this study and in other consulted studies [18,19,20,21], falls within our RIs (7.88–11.90); concentration was 9.77, higher than Smock et al. [19] (8.97, *p* < 0.001), Scott et al. [18] (9.03, *p* < 0.001), and Motta et al. [20] (9.14, *p* < 0.001), but lower than Rodríguez-Cordero et al. [21] (10.10, *p* < 0.001). However, elevated RBC levels may indicate dehydration and predispose to the development of bovine respiratory disease in high-risk newly received calves [2,74]. On the other hand, RDW%, represents the coefficient of variation of the RBCs volume distribution (size) [37]; both in this study and in consulted publications [18,21], falls within our RIs (19.11–30.13); the value was 26.40, similar to Rodriguez-Cordero et al. [21] (26.00, *p* > 0.05), but lower than Scott et al. [18] (27.56, *p* < 0.05). Regarding MCV, provide information about the volume or size of individual RBCs [37]; RIs were generated (29.10–41.80), and the mean was 35.12, higher than that observed by Rodriguez-Cordero et al. [21] (34.10, *p* < 0.001), but lower than that described by Motta et al. [20] (36.68, *p* < 0.001), Scott et al. [18] (38.84, *p* < 0.001), and Smock et al. [19] (40.39, *p* < 0.001).

The function of HGB is to transport oxygen and carbon dioxide throughout the body; therefore, blood’s ability to transport oxygen is determined by the HGB concentration [19]. Although decreased HGB indicates anemia [37,75], values presented in this study and in the compared publications [18,19,20,21] are within our RIs for high-risk newly received calves (9.40–14.40); the concentration was 12.05, higher than Motta et al. [20] (11.26, *p* < 0.05) and Rodríguez-Cordero et al. [21] (11.60, *p* < 0.05), but lower than Smock et al. [19] (12.37, *p* < 0.001) and Scott et al. [18] (12.49, *p* < 0.001). As mentioned, decreased HGB causes symptoms of anemia, and one of the most common causes may be iron deficiency [37,75]; although it also indicates a lack of amino acids, vitamins (especially vitamins B_12_, E, folic acid, and niacin), and/or minerals [76]. On the other hand, MCH quantifies the amount of HGB per RBCs [37]; RIs were generated (10.60–14.4), and the mean was 12.36, similar to Motta et al. [20] (12.37, *p* > 0.05), higher than that observed by Rodríguez-Cordero et al. [21] (11.50, *p* < 0.001), but lower than Scott et al. [18] (13.85, *p* < 0.001), and Smock et al. [19] (13.86, *p* < 0.001). Regarding MCHC, it indicates the amount of HGB per unit volume (correlates the HGB content with the volume of the cell) [37]; RIs were generated (30.58–38.93), and the mean was 35.35, higher than Motta et al. [20] (33.74, *p* < 0.001), Rodríguez-Cordero et al. [21] (34.00, *p* < 0.001), and Smock et al. [19] (34.38, *p* < 0.001), but lower than Scott et al. [18] (34.38, *p* < 0.001).

HCT% or packed cell volume is a measure of the proportion of the total blood volume, which is comprised of RBC and WBC, and is closely related to dehydration [37]; this is a common condition in high-risk newly received calves, which can increase HCT% levels [36,65]. Concentrated HCT% values could indicate anemia, hemorrhages, RBC destruction, or nutritional deficiencies [27,76]; on the other hand, higher values could indicate dehydration [70] caused by diarrhea, erythrosis, and polycythermia vera, and low HCT% can result from vitamin or mineral deficiencies [27]. HCT% in this study and other publications [18,19,20,21], falls within our RIs (26.65–41.93); the value was 34.27, similar to Rodriguez-Cordero et al. [21] (34.30, *p* > 0.05), but higher than Motta et al. [20] (33.35, *p* < 0.05), and lower than Scott et al. [18] (35.00, *p* < 0.01) and Smock et al. [19] (36.08, *p* < 0.001). Richeson et al. [2] and Oosthuysen et al. [77] reported that high-risk newly received calves, subjected to prolonged periods of water deprivation and transportation, suffer dehydration, which is confirmed with HCT% over RIs; and to reverse this dehydration, it is necessary to consume water freely for more than 24 h after arrival [78].

## 4. Conclusions

This study contributes to visualization of RIs for different enzymic activity, metabolites, and hematological responses in high-risk beef calves during their initial period of arriving into feedlot. For some parameters, the means of our study and those of published reports were acceptably close, but not for others. The conditions in which the RIs were generated are representative of the situations that generally occur in Mexican and US feedlot systems with this type of cattle. Therefore, this information can be useful for veterinary clinical practice, as well as for research related to the health and welfare of high-risk beef calves during their initial period of arriving into feedlot under those systems. 

## Figures and Tables

**Table 1:** Descriptive statistics and reference intervals (RIs) ^1^ for enzymic activity and metabolites from high-risk newly received calves’ “clinical health” (*n* = 320), sampled on day 0, 14, 28, 42, and 56.

Item ^2^	Descriptive Statistics						95% Reference Intervals (RIs)			
	Mean	SD ^3^	CV ^4^	Median	Min	Max	Lower Limit of RIs (90% CI) ^5^		Upper Limit of RIs (90% CI)	
ALP, UI	209.70	107.00	51.03	197.00	14.00	562.00	14.40	14.00–57.00	469.60	427.00–539.00
GGT, UI	17.00	8.90	52.35	14.00	10.00	62.00	10.00	10.00–10.00	47.90	33.00–61.00
AST, UI	68.80	21.90	31.83	65.00	32.00	168.00	38.90	37.30–40.60	124.20	115.50–133.00
ALT, UI	26.50	7.30	27.55	25.00	16.00	65.00	16.60	16.20–17.20	44.40	42.00–47.20
TP, g/dL	6.22	0.83	13.34	6.30	4.00	8.00	4.40	4.18–4.61	7.71	7.59–7.83
ALB, g/dL	2.97	0.50	16.84	3.00	1.50	4.40	1.90	1.60–2.10	3.70	3.70–4.30
GLO, g/dL	3.18	0.50	15.72	3.20	2.00	4.30	2.20	2.10–2.30	4.11	4.10–4.30
ALB/GLO ratio	0.94	0.17	18.09	0.92	0.58	1.65	0.68	0.64–0.70	1.32	1.25–1.53
BUN, mg/dL	11.08	2.31	20.85	10.80	6.30	17.40	6.91	6.50–7.20	16.10	15.60–17.10
CRE, mg/dL	0.81	0.20	24.69	0.78	0.48	1.42	0.52	0.49–0.55	1.35	0.49–1.41
TBIL, mg/dL	0.34	0.29	85.29	0.20	0.12	2.00	0.20	0.20–0.20	1.30	1.10–1.80
TCHO, mg/dL	78.60	22.00	27.99	75.00	50.00	148.00	50.00	50.00–50.00	127.70	122.00–129.00
TG, mg/dL	17.00	6.90	40.59	16.00	10.00	36.00	10.00	10.00–10.00	33.00	31.00–35.00
Ca, mg/dL	10.28	1.42	13.81	10.50	6.60	13.40	7.12	6.54–7.38	12.50	12.54–12.88
GLU, mg/dL	89.00	22.50	25.28	92.00	20.00	146.00	26.10	20.00–39.00	126.00	121.00–140.00
Na^+^, mEq/L	126.30	12.10	9.58	128.00	89.00	149.00	98.20	94.00–104.00	143.00	141.00–149.00
K^+^, mEq/L	4.93	1.21	24.54	4.70	2.80	9.20	3.11	3.00–3.40	8.59	7.90–8.80
Cl^−^, mEq/L	90.80	9.80	10.79	91.00	70.00	119.00	71.10	70.00–74.00	109.00	107.00–115.00

^1^ Reference intervals determined with Reference Value Advisor Software (v.2.1) freeware (RefValAdv), an add-in program in Excel (Office 365, Microsoft, Redmond, WA, USA) [31], using the nonparametric method. ^2^ ALP = alkaline phosphatase, GGT = gamma glutamyltransferase, AST = aspartate aminotransferase, ALT = alanine aminotransferase, TP = total protein, ALB = albumin, GLO = globulins, BUN = blood urea nitrogen, CRE = creatinine, TBIL = total bilirubin, TCHO = total cholesterol, TG = triglycerides, Ca = calcium, GLU = glucose, Na^+^ = sodium, K^+^ = potassium, Cl^−^ = chlorine. ^3^ SD = standard deviation. ^4^ CV = coefficient of variation. ^5^ CI = confidence interval.

**Table 2:** Comparison of means of high-risk newly received calves “clinical health” for enzymic activity and metabolites.

Item ^1^	Current Research ^2^	Means of Different Publications ^3^		
		Rodríguez-Cordero et al. [21]	Crawford et al. [17]	Smock et al. [19]
ALP, UI	14.40–469.60 (209.70 ± 107.00)	379.40 ± 37.33 ***	-	111.36 ± 7.81 ***
GGT, UI	10.00–47.90 (17.00 ± 8.90)	16.60 ± 2.06	-	-
AST, UI	38.90–124.20 (68.80 ± 21.90)	74.70 ± 7.61 ***	-	69.38 ± 3.20
ALT, UI	16.6–44.4 (26.50 ± 7.30)	-	16.92 ± 1.39 ***	19.55 ± 0.76 ***
TP, g/dL	4.40–7.71 (6.22 ± 0.83)	5.90 ± 0.22 ***	-	6.96 ± 0.09 ***
ALB, g/dL	1.90–3.70 (2.97 ± 0.50)	3.90 ± 0.50 ***	-	2.45 ± 0.05 ***
GLO, g/dL	2.20–4.11 (3.18 ± 0.50)	3.10 ± 0.20 *	-	4.50 ± 0.10 ***
BUN, mg/dL	6.91–16.10 (11.08 ± 2.31)	11.30 ± 0.49	8.67 ± 0.54 ***	8.22 ± 0.30 ***
CRE, mg/dL	0.52–1.35 (0.81 ± 0.20)	0.75 ± 0.028 ***	1.20 ± 0.07 ***	1.19 ± 0.04 ***
TBIL, mg/dL	0.20–1.30 (0.34 ± 0.29)	0.23 ± 0.018 ***	-	0.29 ± 0.008 **
TCHO, mg/dL	50.00–127.70 (78.60 ± 22.00)	65.50 ± 4.02	-	-
TG, mg/dL	10.00–33.00 (17.00 ± 6.90)	35.10 ± 5.97 ***	-	-
Ca, mg/dL	7.12–12.50 (10.28 ± 1.42)	10.10 ± 1.18 *	-	10.27 ± 0.09
GLU, mg/dL	26.10–126.00 (89.00 ± 22.50)	106.40 ± 7.31 ***	90.17 ± 3.38	94.74 ± 3.06 ***
Na^+^, mEq/L	98.20–143.00 (126.30 ± 12.10)	121.20 ± 1.90 ***	140.75 ± 0.72 ***	135.82 ± 0.31 ***
K^+^, mEq/L	3.11–8.59 (4.93 ± 1.21)	4.4 ± 0.10 ***	5.70 ± 0.13 ***	5.20 ± 0.07 ***
Cl^−^, mEq/L	71.10–109.00 (90.80 ± 9.80)	85.7 ± 1.65 ***	96.88 ± 0.69 ***	94.81 ± 0.28 ***

^1^ ALP = alkaline phosphatase, GGT = gamma glutamyltransferase, AST = aspartate aminotransferase, ALT = alanine aminotransferase, TP = total protein, ALB = albumin, GLO = globulins, BUN = blood urea nitrogen, CRE = creatinine, TBIL = total bilirubin, TCHO = total cholesterol, TG = triglycerides, Ca = calcium, GLU = glucose, Na^+^ = sodium, K^+^ = potassium, Cl^−^ = chlorine. ^2^ Reference Intervals, means, and standard deviation calculated in current research. ^3^ Means and standard error of the mean. *t*-test * *p* < 0.05, ** *p* < 0.01, *** *p* < 0.001 in this publication versus the current investigation.

**Table 3:** Descriptive statistics and reference intervals (RIs) ^1^ for platelets and white and red blood cells from high-risk newly received calves “clinical health” (*n* = 320), sampled on day 0, 14, 28, 42, and 56 (Method: non-parametric).

Item ^2^		Descriptive Statistics					95% Reference Intervals (RIs)			
	Mean	SD ^3^	CV ^4^	Median	Min	Max	Lower limit of RIs (90% CI) ^5^		Upper limit of RIs (90% CI)	
WBC, ×10^3^ cells/μL	9.65	2.62	27.15	9.70	3.50	15.70	4.60	4.20–5.20	15.20	14.20–15.60
LYM, ×10^3^ cells/μL	5.87	1.60	27.26	5.90	1.60	9.80	2.60	2.10–3.10	9.00	8.60–9.50
LYM, %	59.20	9.48	16.01	59.90	13.20	84.50	33.60	27.30–41.70	74.61	72.90–84.50
MON, ×10^3^ cells/μL	0.83	0.26	31.33	0.80	0.20	1.40	0.30	0.20–0.40	1.40	1.30–1.40
MON, %	8.28	1.50	18.12	8.10	3.50	13.50	5.54	5.40–6.40	12.00	11.10–13.10
GRA, ×10^3^ cells/μL	3.33	1.42	42.64	3.10	0.20	7.90	1.10	0.70–1.50	6.74	6.00–7.10
GRA, %	35.30	14.82	41.98	32.10	10.10	86.80	17.51	14.70–20.30	85.57	80.30–86.30
PLT, ×10^3^ cells/μL	239.80	90.70	37.82	224.00	69.00	470.00	91.20	70.00–107.00	444.90	414.00–470.00
MPV, fL	7.22	0.97	13.43	7.10	6.00	15.20	6.14	6.10–6.20	9.08	8.30–10.20
RBC, ×10^6^ cells/μL	9.77	1.07	10.95	9.75	4.97	12.42	7.88	7.37–8.08	11.90	11.56–12.20
RDW, %	26.40	2.54	9.62	26.80	13.70	31.30	19.11	17.70–20.20	30.13	29.30–30.70
HGB, g/100 mL	12.05	1.31	10.87	12.00	7.20	15.70	9.40	9.10–9.90	14.40	14.10–15.20
HCT, %	34.27	3.92	11.44	34.30	17.50	49.90	26.65	25.80–28.70	41.93	40.90–43.40
MCV, fL	35.12	3.10	8.83	34.80	27.50	44.90	29.10	27.50–29.90	41.80	40.30–42.80
MCH, pg	12.36	1.03	8.33	12.40	10.50	15.70	10.60	10.60–10.80	14.40	14.10–15.00
MCHC, g/dL	35.35	1.98	5.60	35.50	29.00	41.50	30.58	30.00–31.10	38.93	38.30–42.00

^1^ Reference intervals determined with Reference Value Advisor Software (v.2.1) freeware (RefValAdv), an add-in program in Excel (Office 365, Microsoft, Redmond, WA, USA) [31], using the nonparametric method. ^2^ WBC = total white blood cells, LYM = lymphocytes, LYM% = lymphocytes%, MON = monocytes, MON% = monocytes%, GRA = granulocytes, GRA% = granulocytes%, PLT = platelets, MPV = mean platelet volume, RBC = red blood cells, RDW% = red blood cells distribution width test%, HGB = hemoglobin, HCT = hematocrit, MCV = mean corpuscular volume, MCH = mean corpuscular hemoglobin, MCHC = mean corpuscular hemoglobin concentration. ^3^ SD = standard deviation. ^4^ CV = coefficient of variation. ^5^ CI = confidence interval.

**Table 4:** Comparison of means of high-risk newly received calves “clinical health” for platelets and white and red blood cells.

Item ^1^	Current Research ^2^	Means of Different Publications ^3^			
		Motta et al. [20]	Rodríguez-Cordero et al. [21]	Scott et al. [18]	Smock et al. [19]
WBC, ×10^3^ cells/μL	4.60–15.20 (9.65 ± 2.62)	-	8.90 ± 0.54 ***	7.32 ± 1.29 ***	13.03 ± 0.72 ***
LYM, ×10^3^ cells/μL	2.60–9.00 (5.87 ± 1.60)	-	5.10 ± 0.31 ***	-	6.79 ± 0.53 ***
LYM, %	33.60–74.61 (59.20 ± 9.48)	-	54.00 ± 1.77 ***	51.63 ± 11.92 ***	53.17 ± 1.19 ***
MON, ×10^3^ cells/μL	0.30–1.40 (0.83 ± 0.26)	-	0.70 ± 0.06 ***	-	1.70 ± 0.05 ***
MON, %	5.54–12.00 (8.28 ± 1.50)	-	7.90 ± 0.34	8.36 ± 4.5	13.43 ± 1.15 ***
GRA, ×10^3^ cells/μL	1.10–6.74 (3.33 ± 1.42)	-	3.10 ± 0.27	-	-
GRA, %	17.51–85.57 (35.30 ± 14.82)	-	38.00 ± 1.70 **	-	-
PLT, ×10^3^ cells/μL	91.20–444.90 (239.80 ± 90.70)	263.81 ± 120.09 ***	243.00 ± 21.08	-	513.70 ± 33.84 ***
MPV, fL	6.14–9.08 (7.22 ± 0.97)	-	7.2 ± 0.25	-	-
RBC, ×10^6^ cells/μL	7.88–11.90 (9.77 ± 1.07)	9.14 ± 1.02 ***	10.10 ± 0.25 ***	9.03 ± 0.68 ***	8.97 ± 0.35 ***
RDW, %	19.11–30.13 (26.40 ± 2.54)	-	26.00 ± 0.48	27.56 ± 3.02 *	-
HGB, g/100 ml	9.40–14.40 (12.05 ± 1.31)	11.26 ± 0.99 *	11.60 ± 0.25 *	12.49 ± 0.91 ***	12.37 ± 0.36 ***
HCT, %	26.65–41.93 (34.27 ± 3.92)	33.35 ± 2.92 *	34.30 ± 0.70	35.0 ± 2.53 **	36.08 ± 0.95 ***
MCV, fL	29.10–41.80 (35.12 ± 3.10)	36.68 ± 2.79 ***	34.10 ± 0.78 ***	38.84 ± 2.85 ***	40.39 ± 0.64 ***
MCH, pg	10.60–14.40 (12.36 ± 1.03)	12.37 ± 0.88	11.50 ± 0.26 ***	13.85 ± 0.81 ***	13.86 ± 0.62 ***
MCHC, g/dL	30.58–38.93 (35.35 ± 1.98)	33.74 ± 0.89 ***	34.00 ± 0.64 ***	35.69 ± 0.98 *	34.38 ± 1.27 ***

^1^ WBC = total white blood cells, LYM = lymphocytes, LYM% = lymphocytes%, MON = monocytes, MON% = monocytes%, GRA = granulocytes, GRA% = granulocytes%, PLT = platelets, MPV = mean platelet volume, RBC = red blood cells, RDW% = red blood cells distribution width test%, HGB = hemoglobin, HCT = hematocrit, MCV = mean corpuscular volume, MCH = mean corpuscular hemoglobin, MCHC = mean corpuscular hemoglobin concentration. ^2^ Reference intervals, means and standard deviation calculated in current research. ^3^ Means and standard error of the mean. *t*-test * *p* < 0.05, ** *p* < 0.01, *** *p* < 0.001 in this publication versus the current investigation.

## Data Availability

The information published in this study is available on request from the corresponding author.
